# Constitutive *BRCA1* Promoter Hypermethylation Can Be a Predisposing Event in Isolated Early-Onset Breast Cancer

**DOI:** 10.3390/cancers11010058

**Published:** 2019-01-09

**Authors:** Jacopo Azzollini, Chiara Pesenti, Sara Pizzamiglio, Laura Fontana, Carmela Guarino, Bernard Peissel, Maddalena Plebani, Silvia Tabano, Silvia Maria Sirchia, Patrizia Colapietro, Roberta Villa, Biagio Paolini, Paolo Verderio, Monica Miozzo, Siranoush Manoukian

**Affiliations:** 1 Unit of Medical Genetics, Department of Medical Oncology and Hematology, Fondazione IRCCS Istituto Nazionale dei Tumori, 20133 Milan, Italy; jacopo.azzollini@istitutotumori.mi.it (J.A.); bernard.peissel@istitutotumori.mi.it (B.P.); roberta.villa@istitutotumori.mi.it (R.V.); 2 Department of Pathophysiology & Transplantation, Università degli Studi di Milano; Fondazione IRCCS Ca’ Granda Ospedale Maggiore Policlinico, 20122 Milano, Italy; chiara.pesenti@unimi.it (C.P.); laura.fontana@unimi.it (L.F.); silvia.tabano@unimi.it (S.T.); patrizia.colapietro@unimi.it (P.C.); monica.miozzo@unimi.it (M.M.); 3 Unit of Bioinformatics and Biostatistics, Department of Applied Research and Technological Development, Fondazione IRCCS Istituto Nazionale dei Tumori, 20133 Milan, Italy; sara.pizzamiglio@istitutotumori.mi.it (S.P.); maddalena.plebani@istitutotumori.mi.it (M.P.); paolo.verderio@istitutotumori.mi.it (P.V.); 4 Immunohematology & Transfusion Medicine Service, Fondazione IRCCS Istituto Nazionale Tumori, 20133 Milan, Italy; carmela.guarino@istitutotumori.mi.it; 5 Medical Genetics, Department of Health Sciences, Università degli Studi di Milano, 20142 Milano, Italy; silvia.sirchia@unimi.it; 6 Department of Pathology and Laboratory Medicine, Fondazione IRCCS Istituto Nazionale dei Tumori, 20133 Milan, Italy; biagio.paolini@istitutotumori.mi.it

**Keywords:** breast cancer, epigenetics, *BRCA1*, promoter methylation

## Abstract

Early age at onset of breast cancer (eoBC) is suggestive of an increased genetic risk. Although genetic testing is offered to all eoBC-affected women, in isolated cases the detection rate of pathogenic variants is <10%. This study aimed at assessing the role of constitutive promoter methylation at BC-associated loci as an underlying predisposing event in women with eoBC and negative family history. Promoter methylation at 12 loci was assessed by the MassARRAY technology in blood from 154 *BRCA1/2* negative patients with eoBC and negative family history, and 60 healthy controls. Hypermethylation was determined, within each promoter, by comparing the patient’s mean methylation value with thresholds based on one-sided 95% bootstrap confidence interval of the controls’ mean. Three patients had hypermethylated results, two at *BRCA1* and one at *RAD51C*. Analyses on tumor tissue from the patient exceeding the highest threshold at *BRCA1* revealed a mean methylation >60% and loss of heterozygosity at chromosome 17q. The patient hypermethylated at *RAD51C* showed low methylation in the tumor sample, ruling out a role for methylation-induced silencing in tumor development. In isolated eoBC patients, *BRCA1* constitutive promoter methylation may be a predisposing event. Further studies are required to define the impact of methylation changes occurring at BC-predisposing genes and their role in tumorigenesis.

## 1. Introduction

Breast cancer (BC) is the most common malignancy and the main cause of cancer-related deaths in women worldwide [1]. While most cases occur after menopause, BC is ranked first in terms of incidence and mortality also among women in the age range of 20–39 years [2]. In Italy, BC represents 41% of all cancer cases in women aged <50 years, with increasing incidence rates recorded in the last years [3].

The risk of developing BC is influenced by multiple factors, including genetic, lifestyle and environmental factors. Although only 5–10% of all cases present a strong genetic predisposition, genetic factors are expected to play a role in up to 30% of BCs [4,5]. Early age at onset, regardless of family history (FH), is considered to be suggestive of an underlying genetic susceptibility and is one of the most common features that prompt genetic testing.

Among known genetic susceptibility factors, the major genes *BRCA1* and *BRCA2* account for 20–30% of all suspected hereditary BC (HBC) and harbor most of the currently identifiable high-risk variants. The detection rate (DR) of *BRCA1*/*BRCA2* pathogenic variants depends on the characteristics of probands/families. Although a young age at diagnosis is commonly associated with a higher probability of carrying pathogenic variants, several studies highlighted that the DR might be even lower than 10% in patients with early onset BC (eoBC) and negative FH [6,7,8,9,10]. Other high-penetrance variants, accounting for about 1% of HBCs, are found in the *TP53*, *PTEN*, *STK11*, *CDH1* and *PALB2* genes. Moderate-penetrance variants have been described in about 5% of cases, although their contribution to HBC appears to be limited [11,12,13,14]. In addition, roughly 18% of the familial relative risk has been attributed to the combined risk conferred by over 170 common variants, identified through large genome-wide association studies [15,16]. However, to date the presence of rare high-risk sequence variants or common low-risk alleles alone does not explain the majority of HBC cases.

In order to identify other putative causes of increased susceptibility to BC, which might also underlie a fraction of both familial and sporadic eoBC cases, alternative pathomechanisms have been sought. An emerging phenomenon, which has also been associated with increased risk of BC and other types of cancer, consists of epigenetic dysregulation, such as hypermethylation at specific regions, resulting in the inactivation of tumor suppressor genes [17,18]. Hypermethylation events, usually involving gene promoters, have been shown to act as a “first hit” in the Knudson model by down-regulating gene transcription, with a functional effect similar to that of inactivating sequence variants. These events are found either confined to the tumor or might also involve other tissues, including blood. In the latter case, aberrant epigenetic processes are hypothesized to occur during early developmental stages [19,20,21,22].

Previous studies, which analyzed peripheral blood of BC-affected individuals, documented constitutive hypermethylation at *BRCA1* and other loci. In particular, Hansmann et al. identified constitutive hypermethylation at *BRCA1* and *RAD51C* in 1.4% (9/641) and 0.5% (3/641) of high risk patients respectively [23]. Furthermore, the authors reported a prevalence of promoter hypermethylation of 5.5% (2/37) in women with eoBC and negative FH, which was significantly higher compared with other risk groups, thus suggesting that this event might be more frequent in these patients.

Since young BC-affected women with negative FH in our population show a very low DR (<8%) at routine genetic testing [10], a possible explanation would be that epigenetic dysregulation acts as an alternative predisposing event in a subset of these patients. In order to test this hypothesis, we carried out a quantitative methylation analysis of CpG islands at promoter regions of 12 known cancer genes (*BRCA1*, *BRCA2*, *ATM*, *CDH1*, *FANCM*, *STK11*, *NBN*, *PALB2*, *PTEN*, *RAD51C*, *RECQL* and *TP53*), on a large cohort of 154 unrelated women affected with eoBC and selected for negative FH.

## 2. Results

### 2.1. CpGs Selection

In the experiment implemented on 8 healthy donors, a total of 232 CpGs from the 12 investigated promoters were analyzed. Among those, 81 CpGs were excluded according to the EpyTYPER warning remarks (“D” or “OL”, overlaps in mass between other fragments or “H/L_mass”, too high/low mass) and 32 CpGs were excluded based on the quality-filtering criteria. As a result, 119 CpGs were selected for the subsequent analysis. All these CpGs had a success rate > 80% across all the samples (154 cases + 60 controls) and standard deviation <0.1 in control samples.

### 2.2. Analysis of Promoter Methylation in Control Samples

**Figure 1:**
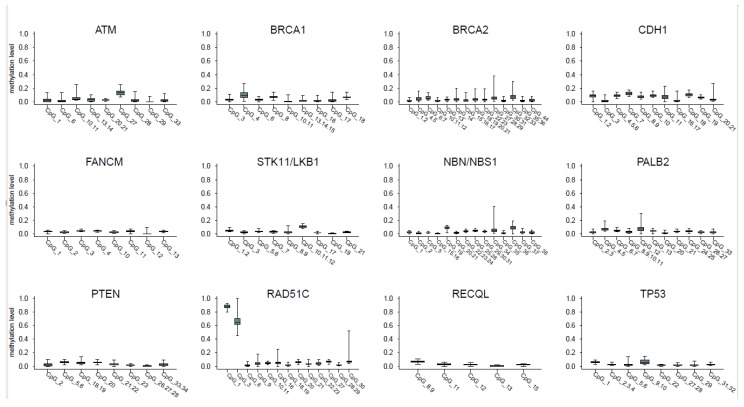
distribution of blood methylation levels of selected CpGs within each promoter. Each panel reports the box plot of the blood methylation levels at the selected CpGs within a single promoter. The boxes indicate the 25th and 75th percentiles of the distribution. The horizontal line inside the box indicates the median and the whiskers indicate the extreme (max and min) values of the blood methylation levels.

**Figure 2:**
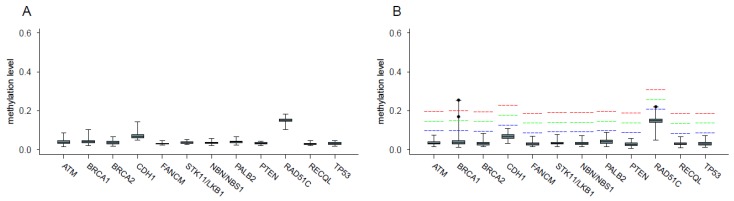
distribution of the mean methylation levels at each promoter in blood samples from the 60 controls (panel **A**) and 154 patients (panel **B**); the red, green and blue dashed lines indicate the thresholds used for the identification of hypermethylated cases obtained by adding the prefixed value of 0.15, 0.1 and 0.05 respectively to the one-sided 95% bootstrap confidence interval of the controls’ mean; the dots indicate mean methylation level of the three hypermethylated cases.

Figure 1 shows the distribution of blood methylation levels of the selected CpGs within each promoter; panel A of Figure 2 describes the distribution of the mean methylation level of each promoter in blood samples from the 60 controls.

In all the investigated promoters, the level of methylation (median value) of most CpGs showed <0.1 as expected, with the exception of a single CpG in *CDH1*, *ATM* and *STK11*, which showed a median methylation value in the range 0.1–0.15, and two CpGs in *RAD51C*, which were consistently hypermethylated in all samples (median values >0.8 and >0.6, respectively; raw data provided in Table S1).

### 2.3. Analysis of Promoter Methylation in Patients and Identification of Hypermethylated Cases

Pathological features of the analyzed cohort of patients are reported in Table 1.

Panel B of Figure 2 shows the distribution of the mean blood methylation levels at each promoter in samples from the 154 patients (raw data provided in Table S2). Among these individuals, three (1.8%) showed mean blood methylation levels exceeding the threshold by at least +0.05 at a single gene promoter. Patient 1 exceeded the threshold by +0.15 at the *BRCA1* promoter, with mean methylation value of 0.25. The analysis of the corresponding patient’s tumor tissue revealed a mean methylation >0.60 (Table S3). The *BRCA1* promoter assay specifically used for FFPE BC was also tested on normal breast tissue derived from breast reduction surgery of four healthy controls, to establish the normal methylation level in FFPE tissues, which was <0.1. No variants with a possible cis-acting regulator effect on promoter methylation were identified by means of Sanger sequencing, in the region 500 bp upstream of the translation starting site.

Patient 2 exceeded the threshold by +0.05 at the *RAD51C* promoter in blood (mean methylation level 0.22). However, the mean *RAD51C* methylation, observed in the patient’s tumor (0.13), was lower than that observed in blood and within the normal range with respect to normal breast tissues (0.11–0.16) (Table S3). Patient 3, with mean *BRCA1* promoter methylation of about 0.17, exceeded the threshold by +0.10. No tumor tissue was available for further evaluation. All other patients did not exceed the threshold by at least +0.05 in any of the investigated promoters.

### 2.4. Assessment of Loss of Heterozygosity (LOH)

In order to assess whether a complete loss of BRCA1 underpinned tumor development in patient 1 and explained the *BRCA1* promoter methylation above 0.50 detected in the tumor tissue, the loss of heterozygosity (LOH) at chromosome 17 was investigated.

**Figure 3:**
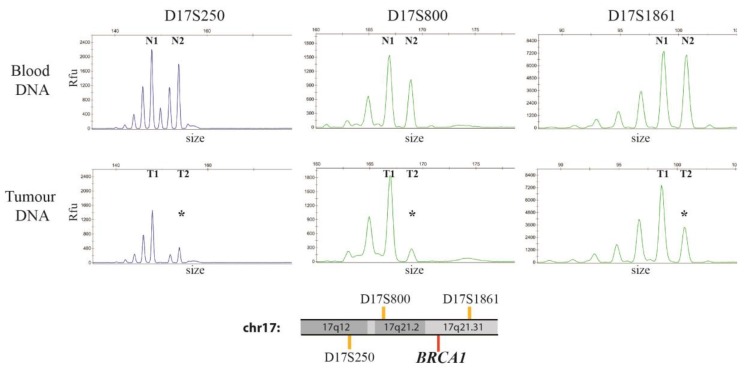
Electropherograms of the three informative STRs in blood and tumor DNA from patient 1; peaks profiles of STRs D17S250, D17S800 and D17S1861 obtained on blood DNA (top panels) and tumor DNA (bottom panels) from Patient 1; x-axis indicates the size in base pairs of each STR while the y-axis refers to the Relative Fluorescence Units (Rfu) of the detected signal, which is directly proportional to the number of copies of each allele. The three STRs had heterozygous results in blood DNA and were thus informative for LOH analysis; the two alleles identified in blood DNA are indicated by N1 and N2 for each STR, while T1 and T2 are the corresponding alleles in tumor DNA. The lost allele for each STR in tumor DNA (T2 for all STRs) is indicated by an asterisk; LOH was confirmed applying the formula on the peak heights described in the methods, which provided values of 0.25, 0.2 and 0.46 respectively (value for LOH <0.50). Genomic location of the three STRs (orange lines) with respect to *BRCA1* (17q21.31, red line) are depicted beneath the electropherograms.

Five Short Tandem Repeats (STRs: D17S250, D17S800, D17S951, D17S1861 and D17S932) at chromosome 17 were analyzed in tumor samples and normal tissue from patient 1. Three STRs showed heterozygous results in DNA from normal tissue and thus were informative for the analysis (D17S250, D17S800 and D17S1861). The presence of LOH was assessed by comparing the signal from the two alleles of the three STRs in blood and tumor DNA. Figure 3 shows a reduced signal intensity of the second allele at each informative STR in the tumor sample. 

The calculated allele/peak ratio of the three STRs consistently confirmed LOH in the tumor, indicating the presence of a large deletion encompassing *BRCA1* (17q12–17q21.31). 

### 2.5. Relationship between Methylation Levels, Age at Blood Withdrawal and Chemotherapy

In order to determine whether oncological treatments influenced promoter methylation at the selected loci, we compared for each promoter the mean methylation of the selected CpGs between patients who underwent blood withdrawal during or following chemotherapy and those who did not undergo chemotherapy before blood withdrawal. Age at blood withdrawal, as an additional factor influencing DNA methylation was also investigated. For all promoters, no statistically significant association was observed either between mean methylation level and age at blood withdrawal in both controls and cases (Figure S1, panels A and B) or between methylation and systemic treatments (Figure S1, panel C).

### 2.6. Evaluation of BRCA1 Expression Levels

**Figure 4:**
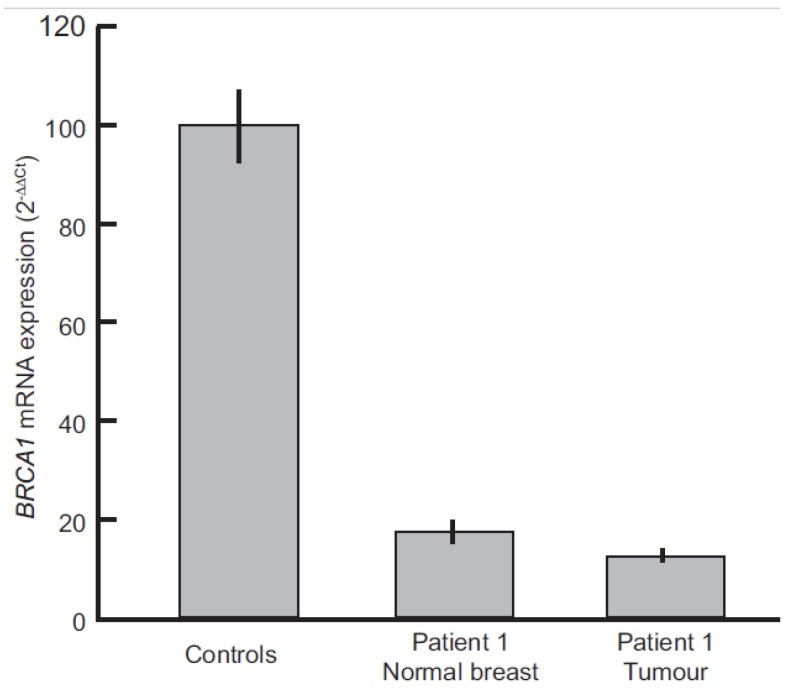
BRCA1 mRNA levels in Patient 1 tumor tissue and normal breast vs. breast tissue from healthy controls (*n* = 2), as determined by real-time PCR. Bars represent mean expression values and vertical lines indicate the standard deviation between at least two independent experiments.

To quantitatively measure the effect of promoter methylation on *BRCA1* mRNA expression in patient 1, Real-time PCR was performed. Figure 4 shows the *BRCA1* mRNA levels of normal breast and tumor tissue from patient 1. Compared with normal breast tissue from two healthy women, *BRCA1* expression levels in patient 1 were very low in both normal breast and tumor samples, with a reduction of 82.6% and 87.6% respectively (Figure 4).

## 3. Discussion

Promoter methylation is a well-established mechanism leading to gene silencing. Although a role as a first hit in BC development has been described, usually occurring at tumor suppressor genes, its contribution as a constitutive susceptibility factor is yet to be fully elucidated.

Previous studies, focusing mainly on the promoter of *BRCA1* and a few other high- or moderate-penetrance genes, reported an association between methylation levels in peripheral blood and risk of BC [24,25,26,27,28]. Most of these studies though considered heterogeneous cohorts of patients, including sporadic or familial BC and/or ovarian cancer (OC), triple-negative BC (TNBC), eoBC and male BC, and reported a highly variable frequency of hypermethylation events in both patients and healthy individuals.

As concerns *BRCA1* promoter hypermethylation detected in blood, the estimated prevalence ranges between 10% and 63% in BC patients and between 3% and 63% in healthy controls [25,28,29]. This great variability, which burdens the identification of hypermethylation with a potential functional effect, might be due to multiple factors, such as genomic location and CpGs selection, cut-offs set to define hypermethylation and techniques used for the analysis. In particular, techniques that analyze only a single or a limited number of CpGs, which are then used to infer the methylation level of a larger region, might provide misleading results. Our study highlighted that among contiguous CpGs within the same genomic locus, some display highly variable methylation levels even among healthy individuals and are not representative of the global promoter methylation. Moreover, CpGs showing both inter-experimental and inter-individual stable methylation levels, might display pronounced differences compared with other CpGs within the same region.

Since the frequency of promoter hypermethylation might also be influenced by the cohort of enrolled individuals, we decided to focus on women affected by eoBC and selected for negative FH (defined as no additional BC or OC cases up to the third degree of kinship). The analysis of isolated eoBC cases was prompted both by the very low detection rate of *BRCA1/BRCA2* pathogenic variants, despite their a priori increased genetic risk, and by the hypothesis of a higher hypermethylation frequency in this type of patient, as suggested by Hansmann et al. [23]. In principle, a constitutive methylation event occurring during early development, rather than the presence of an inheritable sequence variant, is consistent with a higher risk involving only one individual within a family.

By analyzing 154 cases, representing the largest cohort reported to date of isolated eoBC cases investigated for promoter methylation at multiple loci, we identified three cases with higher methylation levels compared with healthy individuals, two at the *BRCA1* promoter (exceeding the threshold by +0.15 and +0.10 respectively) and one at the *RAD51C* promoter (exceeding the threshold by +0.05). The patient with the highest methylation at *BRCA1*, who developed a triple negative high-grade invasive ductal carcinoma at the age of 31 years, showed a mean blood methylation level of 0.25, possibly due to the presence of a constitutively hypermethylated allele. An alternative theory would be that the level of methylation detected in blood from our patient reflected the presence of tumor DNA. However, the size of cell-free DNA fragments is about 150 bp, considerably shorter than our PCR amplicons and thus not detectable by the assays used for the analysis. As for concerns circulating tumor cells (CTCs), the density of which is as low as 1 cell per 1 × 10^9^ blood cells in patients with metastatic cancer, their putative contribution would not justify the high methylation level of patient 1 [30]. Moreover, the chances of detecting tumor DNA in blood seem to increase with tumor stages, and thus with tumor volume [31]. Since our patient was diagnosed with a pT1b tumor without clinical or pathological signs of metastasis (pN0sn), this hypothesis is likely to be very remote.

Subsequent analyses on tumor tissue revealed *LOH* along with mean promoter methylation >0.60. Since previous studies showed that promoter methylation usually occurs as a first step in tumor development [32,33], these findings support the hypothesis of a loss of the unmethylated allele as the second step in patient 1. Accordingly, the analysis of *BRCA1* mRNA revealed decreased expression levels in both normal breast and tumor tissue from patient 1. Moreover, as expected the expression in the tumor sample was lower than that in normal breast, likely due to the presence of LOH. 

In order to rule out the presence of regulatory sequence variants that might determine a *cis*-acting effect on promoter methylation and gene silencing, we performed Sanger sequencing of the *BRCA1* 5′-UTR, which confirmed the absence of variants in the 500 bp upstream of the translation starting site. This phenomenon, usually underlying heritable constitutional epimutations rather than sporadic methylation events, is well characterized for mismatch repair genes [34], and has been recently described for *BRCA1* by Evans et al., who reported a co-segregation of the c.-107A>T variant with *BRCA1* promoter methylation and gene silencing in multiple affected individuals of two families from North West England [35].

Patient 2, who developed an invasive ductal carcinoma (estrogen, progesterone and HER2/neu positive staining, tumor size pT1a, grade II, pN0/1sn) at the age of 31 years and showed hypermethylation exceeding the threshold by +0.05 at *RAD51C*, was also evaluated for promoter methylation in the tumor tissue. The analysis revealed a methylation level within the normal range, thus ruling out a possible role of epigenetic dysregulation in tumor development.

Patient 3, exceeding the threshold by +0.10 at *BRCA1*, developed an invasive ductal carcinoma (estrogen and progesterone negative staining, HER2/neu positive staining, tumor size pT3, grade III, pN7/21) at the age of 28 years. Further analyses on patient 3 could not be carried out, since tumor tissue was not available.

There are a few limitations to our study. First, although the MassARRAY system compared with most other techniques is able to analyze sequences encompassing a larger number of contiguous CpG sites, for some of the investigated promoters the selected CpGs, due to the limited knowledge of the promoters’ structure, might still not be representative of the global promoter methylation. If that were the case, the frequency of hypermethylation events in our cohort would be underestimated. Moreover, the detection rate could have also been influenced by the thresholds used to identify hypermethylated cases. A value of +0.05 with respect to the one-sided 95% bootstrap confidence interval of the controls’ mean might be too conservative and, although patient 2 (+0.05 at *RAD51C*) showed a normal methylation pattern in cancer tissue, we cannot exclude that slighter methylation changes could affect BC risk in other patients. Since hypermethylation in our cohort is assumed to be a somatic mosaic event, methylated alleles could be present at low level in blood DNA, and possibly not detectable by MassARRAY or other available techniques. Consistent with this, a recent study by Böck et al. showed that slight increase in promoter methylation affecting tumor suppressor genes could still determine transcriptional dysregulation in blood, although a direct effect on tumor development could not be ascertained [36]. Second, with respect to the study by Hansmann et al. [23], we did not perform clonal bisulfite sequencing in order to confirm that the hypermethylation detected in blood was allele-specific. Nevertheless, we carried out analyses on tumor tissue that led to the identification of LOH, indicating that the methylation level detected in the tumor reflected the contribution of a single allele. Third, the assessment of methylation levels may be biased by multiple factors, including age, chemotherapy and other environmental and behavioral factors [37,38,39,40]. In regards to age, while several studies demonstrated an increase in methyl-CpGs content at promoter-associated CpG islands [41], it is unlikely that this phenomenon has influenced our conclusions, since blood withdrawal from the three patients showing hypermethylation was carried out respectively at age 31, 33 and 45 years (median age at blood withdrawal 35 years in cases and 41.5 years in controls). Moreover, in our cohort we evaluated both age at blood withdrawal and systemic treatments, which showed no significant effect on promoter methylation at the investigated loci. The effect of other environmental factors on DNA methylation is not well characterized and could not be assessed in our cohort [39].

In conclusion, while constitutive epigenetic derangement appears not to be as frequent in isolated eoBC patients as hypothesized, our findings provide additional elements supporting a role of constitutive *BRCA1* promoter methylation as a factor predisposing to eoBC, in particular of the TN type. On the other hand, they also provide evidence that not all methylation changes involving tumor suppressor genes detected in blood are responsible for tumor pathogenesis. Although the definition of hypermethylation thresholds is often troublesome, we also highlight that a proper selection process of CpGs, based on the analysis of healthy controls, could help facilitate the correct identification of constitutive methylation events. In addition, the analysis of tumor tissue corroborates the role of hypermethylation as a first step. 

## 4. Methods

### 4.1. Study Subjects

Patients were selected among individuals and families who underwent genetic counselling and testing at our Institution. Selection criteria included: women affected with BC, either invasive or ductal carcinoma in situ (DCIS); age at onset before the age of 36 years or before the age of 45 years, in case of documented triple negative phenotype; available and updated information on FH and no additional cases of BC or OC up to the 3rd degree of kinship; no pathogenic variants (including large deletions/duplications) or variants of uncertain significance (VUS) identified by the *BRCA1* and *BRCA2* analysis carried out through sequencing and multiplex ligation-dependent probe amplification (MLPA). Patients, whose reported FH was not considered reliable due to uncertain or insufficient information were excluded from the study.

A total of 154 patients were selected for the analysis on peripheral blood. The median age at onset of BC was 33 years (range 19–44 years); 21 developed a second breast cancer at a median age of 42 years (range 29–68 years). Histopathological features of BC are summarized in Table 1.

Blood samples were consecutively collected also from a control population of 60 women, selected by using age as a matching criterion, among healthy blood donors at the Immunohematology and Transfusion Medicine Service of our Institution. The age at blood withdrawal ranged from 19 to 66 years (median 35) for patients and 21 to 59 years (median 41) for healthy controls.

Furthermore, four Formalin-fixed Paraffin-Embedded (FFPE) samples collected from healthy women, who underwent breast reduction surgery, were used as controls for the methylation and expression analyses.

All participants provided a signed informed consent for the use of their biological samples and data for research purposes. The investigations were conducted in accordance with the Declaration of Helsinki and the study was approved by the Ethics Committee of Fondazione IRCCS Istituto Nazionale dei Tumori (approval code INT 171/15; approval date 28 October 2015).

### 4.2. DNA Extraction and Bisulfite Conversion

Whole blood DNA was extracted through the QIAamp^®^ DNA Mini and Blood Mini Kit (QIAGEN, GmbH, Hilden, Germany) following protocol instructions. FFPE tumor tissue samples, from patients with abnormal methylation levels detected in blood cells, were also retrieved for DNA extraction. In order to reduce the contamination of the tumor component by non-neoplastic cells, manual microdissection was performed on a 7 µm FFPE tissue section previously stained with hematoxylin-eosin. DNA from FFPE tumor and normal samples was extracted with the QIAamp^®^ DNA FFPE Tissue Kit (QIAGEN), according to the protocol instructions.

Bisulfite conversion of genomic DNA from whole blood and FFPE tissues was performed with the EZ DNA Methylation Direct Kit (Zymo Research, Irvine, CA, USA), using 500 ng of DNA for each reaction.

### 4.3. Primer Design and Mass Spectrometry

We selected 12 loci for the study: *BRCA1*, *BRCA2*, *TP53*, *PTEN*, *CDH1*, *PALB2*, *STK11*, *ATM*, *NBN*, *RAD51C*, *RECQL* and *FANCM*. These genes have been either found hypermethylated at promoters in BC patients or their sequence variants have been associated with an increased risk of BC. Promoter regions were selected based on previously published data and in silico prediction of promoter sequences (FirstEF, http://rulai.cshl.org/tools/FirstEF/). For most of the considered genes (i.e., *BRCA1*, *RAD51C*, *ATM*, *CDH1*, *PTEN*, *STK11*, *PALB2*), promoter regions to be investigated were selected based on previous studies, which demonstrated a correlation between hypermethylation and either reduced gene expression or increased risk of BC or both. The other analyzed regions (i.e., *BRCA2*, *TP53*, *NBN*, *RECQL*, *FANCM*) are within sequences with strong promoter activity, but for which fewer functional information related to methylation were available [23,25,42,43,44,45,46,47,48,49,50,51,52]. For each promoter, PCR primers with 5′ T7-promoter tags were designed, using the EpiDesigner software (https://www.epidesigner.com, Agena Bioscience, San Diego, CA, USA) in order to amplify bisulfite-converted 300–500 bp-long genomic sequences including multiple CpGs (range 13–44, median 32). 

The procedure was recently described by Fontana et al. [53]. To avoid preferential amplification of methylated or non-methylated DNA, the primers did not encompass any CpG sites. Further details on primer sequences and targeted genomic regions are described in Table S4. Different pairs of primers, targeting overlapping shorter sequences (about 120–160 bp) at the *BRCA1* and *RAD51C* promoters, were also designed in order to assess the methylation level in FFPE tumor tissues from patients with blood methylation significantly higher compared with control samples (Table S5). In the analysis of the *BRCA1* promoter in FFPE tissues we included only the last 7 CpGs investigated in blood DNA, because no optimal assay could be designed for the upstream region. The *RAD51C* promoter region analyzed on blood DNA was entirely covered by three assays designed for FFPE DNA. After PCR amplification, experiments were carried out following the instruction manual (Epityper User Guide, https://agenacx.com/wp-content/uploads/2017/10/MassARRAY-EpiTYPER-User-Guide.pdf, Agena Bioscience), PCR products were treated with shrimp alkaline phosphatase enzyme (Agena Bioscience) to dephosphorylate unincorporated deoxynucleotide triphosphate. The in vitro transcription and T-cleavage reaction were carried out as described in the instruction manual. Mass spectra were acquired through a MassARRAY mass spectrometer (Agena Bioscience) and analyzed using the EpiTYPER^®^ MassARRAY^®^ software, which provided a quantification of methylated/unmethylated CpGs, with values ranging from 0 to 1.

### 4.4. CpG Selection

An ad hoc experiment with samples from 8 healthy donors (controls) was performed in order to select CpG sites according to quality criteria such as reliability and reproducibility. In this experiment, two independent bisulfite modifications, in duplicate for each sample, were processed for methylation analysis of the 12 investigated promoters. Within and between variability and success rates were considered in order to select reliable CpG sites for the subsequent analysis. Specifically, CpGs with less than 80% of successful measurements or with a standard deviation of 0.1 (10% methylation) and larger were removed. In addition, CpG sites with warning remarks by the EpiTYPER software indicating unreliable data were also excluded.

### 4.5. Statistical Methods

To identify hypermethylated cases, the following procedure was adopted: for each subject (cases and controls) the mean methylation levels of the selected CpGs within each promoter was computed. Starting from the distribution of mean methylation levels of the 60 controls, 1000 bootstrap samples were generated and the one-sided 95% percentile bootstrap confidence interval (bCI) of the controls’ mean was derived [54]. Subsequently, cases with mean methylation exceeding by prefixed values (i.e., 0.05, 0.10 and 0.15) the one-sided 95% bCI were re-analyzed starting from the bisulfite modification step. Cases for which this result was confirmed were classified as hypermethylated. The association between methylation level and chemotherapy status (blood withdrawal during or following chemotherapy vs no chemotherapy before blood withdrawal) was assessed by resorting to univariate logistic regression analysis [55]. The possible correlation between mean methylation level of each promoter and age at blood withdrawal was assessed by the Spearman’s rank correlation coefficient [56].

### 4.6. Evaluation of LOH by STRs Analysis

PCRs were performed using 100 ng of DNA from both tumor and normal breast tissue samples, and the products were analyzed by capillary gel electrophoresis using Gene Mapper software on an ABI 3130XL system (Applied Biosystems, Foster City, CA, USA). Five STRs (D17S250, D17S800, D17S1861, D17S951, D17S932) were used to investigate the presence of deletions involving the *BRCA1* locus on chromosome 17q. Genomic locations of the STRs and primer sequences used for PCRs are provided in Table S6. LOH was assessed on heterozygous STRs, according to the peak-height ratio. In particular, the peak height derived from each allele in the tumor and corresponding normal DNA was compared. The formula (T1/T2)/(N1/N2) was applied to assess the LOH ratio, where T1 and T2 are the peak heights of the alleles detected in the tumor DNA, and N1 and N2 are the peak heights observed in blood DNA. LOH was considered to be present if the ratio was lower than 0.50. For values >1.00, the ratio was converted to 1/[(T1/T2)/(N1/N2)] and LOH was considered to be present if the resulting value was <0.50.

### 4.7. BRCA1 Promoter Sequencing

The two patients, exceeding the methylation threshold at *BRCA1*, were analyzed by Sanger sequencing to check the presence of sequence variants that might determine a *cis*-acting effect on promoter methylation and gene silencing. Primers used for sequencing cover the whole region (c.-516 to c.-24) which was also investigated for methylation profiling: forward primer 5′-AACTGGAGACCTCCATTAGG-3′ and reverse primer 5′-ACCCAGAGCAGAGGGTGAA-3′. PCR was performed with the AmpliTaq Gold DNA polymerase (Thermo Fisher Scientific, Scotland, UK) and sequencing with the Big Dye Terminator v3.1 Cycle sequencing Kit (Thermo Fisher Scientific) on an ABIPRISM 3130XL Genetic Analyzer (Applied Biosystems, Foster City, CA, US).

### 4.8. mRNA Extraction and BRCA1 Expression Quantification by Real-Time PCR

Real-time PCR experiments were performed in order to quantify *BRCA1* mRNA expression levels in normal breast and tumor samples from patient 1 and normal breast tissue from two healthy women, which were also analyzed for methylation levels. Total RNA was extracted from FFPE tissues through the RNeasy FFPE Kit (QIAGEN) following the manufacturer’s instructions and quantified by Nanodrop instrument (Thermo Fisher Scientific) with standard protocol.

An amount of 500 ng of RNA were reverse transcribed by two independent experiments using the High Capacity cDNA Reverse Transcription Kit (Thermo Fisher Scientific) with random hexamers. Real-time PCR experiments were conducted on TaqMan 9700 Realtime PCR system (Applied Biosystems). TaqMan probes were used to detect target (*BRCA1*—assay ID: Hs01556193_m1) and housekeeping (18S rRNA—assay ID: Hs03928990_g1) genes, respectively. Relative gene expression was determined using the DDCt method. Each sample was run in triplicate and the expression value represents the mean among the three replicates.

## 5. Conclusions

The presented data demonstrate that constitutive promoter methylation is not frequently altered in isolated eoBC patients. Nevertheless, *BRCA1* silencing by constitutive methylation might be more frequent in young patients affected with TNBC. Investigations on tumor tissues provided additional elements supporting the role of hypermethylation as a first hit in tumor development. However, they also highlighted that an increased methylation detected in blood might not always underlie tumor development.

Further studies on larger cohorts will help define to what extent constitutive promoter hypermethylation leads to BC (or other type of cancers) and better define a threshold which could be used to identify hypermethylation with potential functional effects.

## Figures and Tables

**Table 1:** Breast cancer (BC) features of the 154 selected patients; presence of distant metastasis, occurrence of second breast cancers and timing of blood withdrawal with respect to systemic chemotherapy are also reported; n.a., information not available.

Breast Cancer Histotype		Ductal	Lobular	Mixed	Other	n.a.	Total
Number of cases		129	2	4	8	11	154
Median Age at diagnosis (range)		32 (19–44)	30; 35	30 (24–35)	34.5 (26–38)	34 (31–40)	33 (19–44)
Grade	I	10 (7.7%)	-	-	-	-	10 (6.5%)
	II	38 (29.5%)	2 (100%)	3 (75%)	2 (25%)	2 (18.2%)	47 (30.5%)
	III	68 (52.7%)	-	1 (25%)	5 (62.5%)	6 (54.5%)	80 (51.9%)
	n.a.	13 (10.1%)	-	-	1 (12.5%)	3 (27.3%)	17 (11.0%)
Tumor size (pT)	Is	4 (3.1%)	-	-	-	-	4 (2.6%)
	1	67 (51.9%)	-	-	3 (37.5%)	-	70 (45.5%)
	2	30 (23.3%)	1 (50%)	3 (75%)	4 (50%)	3 (27.3%)	41 (26.6%)
	3	3 (2.3%)	-	-	-	-	3 (1.9%)
	4	2 (1.6%)	1 (50%)	-	-	-	3 (1.9%)
	n.a.	23 (17.8%)	-	1 (25%)	1 (12.5%)	8 (72.7%)	33 (21.4%)
Lymph Node Metastasis (pN)	+	57 (44.2%)	-	3 (75%)	3 (37.5%)	6 (54.5%)	69 (44.8%)
	−	63 (48.8)	2 (100%)	1 (25%)	5 (62.5%)	2 (18.2%)	73 (47.4%)
	n.a.	9 (7%)	-	-	-	3 (27.3%)	12 (7.8%)
Distant Metastasis (M)	+	8 (6.2%)	-	1 (25%)	2 (25%)	1 (9.1)	12 (7.8%)
	−	121 (93.8%)	2 (100%)	3 (75%)	6 (75%)	10 (90.9%)	142 (92.2%)
Estrogen Receptor (ER staining)	+	80 (62%)	1 (50%)	4 (100%)	5 (62.5%)	5 (45.4%)	95 (61.7%)
	−	45 (34.9%)	1(50%)	-	3 (37.5%)	4 (36.4%)	53 (34.4%)
	n.a.	4 (3.1%)	-	-	-	2 (18.2%)	6 (3.9%)
Progesterone Receptor (PgR staining)	+	57 (44.2%)	2 (100%)	4 (100%)	4 (50%)	5 (45.4%)	72 (46.8%)
	−	65 (50.4%)	-	-	4 (50%)	4 (36.4%)	73 (47.4%)
	n.a.	7 (5.4%)	-	-	-	2 (18.2%)	9 (5.8%)
HER2 staining	+	45 (34.9%)	1 (50%)	-	-	1 (9.1%)	47 (30.5%)
	−	56 (43.4%)	-	1 (25%)	6 (75%)	7 (63.6%)	70 (45.5%)
	n.a.	28 (21.7%)	1 (50%)	3 (75%)	2 (25%)	3 (27.3%)	37 (24.0%)
Triple Negative Phenotype		25 (19.4%)	-	-	3 (37.5%)	3 (27.3%)	31 (20.1%)
2nd Breast Cancer		17 (13.2%)	1 (50%)	2 (50%)	-	1 (9.1%)	21 (13.6%)
2nd Breast Cancer Median age (range)		42 (29–68)	38	50;33	-	38	42 (29–68)
Blood withdrawal (before/after chemotherap)	before CT	37 (28.7%)	-	-	1 (12.5%)	-	38 (24.7%)
	after CT	92 (71.3%)	2 (100%)	4 (100%)	7 (87.5%)	11 (100%)	116 (75.3%)

## Supplementary Materials

The following are available online at http://www.mdpi.com/2072-6694/11/1/58/s1, Figure S1. Evaluation of the relationship between methylation and age and between methylation and systemic treatments. Panel A and B report the Spearman correlation coefficient estimate (diamond) and its 95% Confidence Interval (CI) computed between age at blood withdrawal and methylation level of each promoter in controls (A) and cases (B). Panel C reports the odds ratio estimate (diamond) and the 95% CI obtained for each promoter from the univariate logistic model by considering the chemotherapy status at the time of blood withdrawal as a dependent variable (blood withdrawal during or after chemotherapy vs no chemotherapy before blood withdrawal). Table S1. Methylation levels of selected CpGs in blood DNA samples from 60 healthy controls. Table S2. Methylation levels of selected CpGs in blood DNA samples from 154 isolated eoBC patients. Table S3. Methylation level of selected CpGs at *BRCA1* and *RAD51C* in normal breast samples from four healthy women and in tumor tissue from patients 1 and 2. Table S4. Genomic regions analyzed by MassARRAY on blood DNA. Table S5. Genomic regions analyzed by MassARRAY on tumor DNA from patient 1 (*BRCA1*) and 2 (*RAD51C*). Table S6. Short tandem repeats (STRs) analyzed in blood and tumor DNA from patient 1.
