# Supplementary material for: Constitutive BRCA1 Promoter Hypermethylation Can Be a Predisposing Event in Isolated Early-Onset Breast Cancer
Source: Cancers (Basel). 2019 Jan 9;11(1):58. doi: 10.3390/cancers11010058 (PMC6356733; doi:10.3390/cancers11010058)

Constitutive *BRCA1* Promoter Hypermethylation Can Be a Predisposing Event in Isolated Early-Onset Breast Cancer

Jacopo Azzollini, Chiara Pesenti, Sara Pizzamiglio, Laura Fontana, Carmela Guarino, Bernard Peissel, Maddalena Plebani, Silvia Tabano, Silvia Maria Sirchia, Patrizia Colapietro, Roberta Villa, Biagio Paolini, Paolo Verderio, Monica Miozzo and Siranoush Manoukian


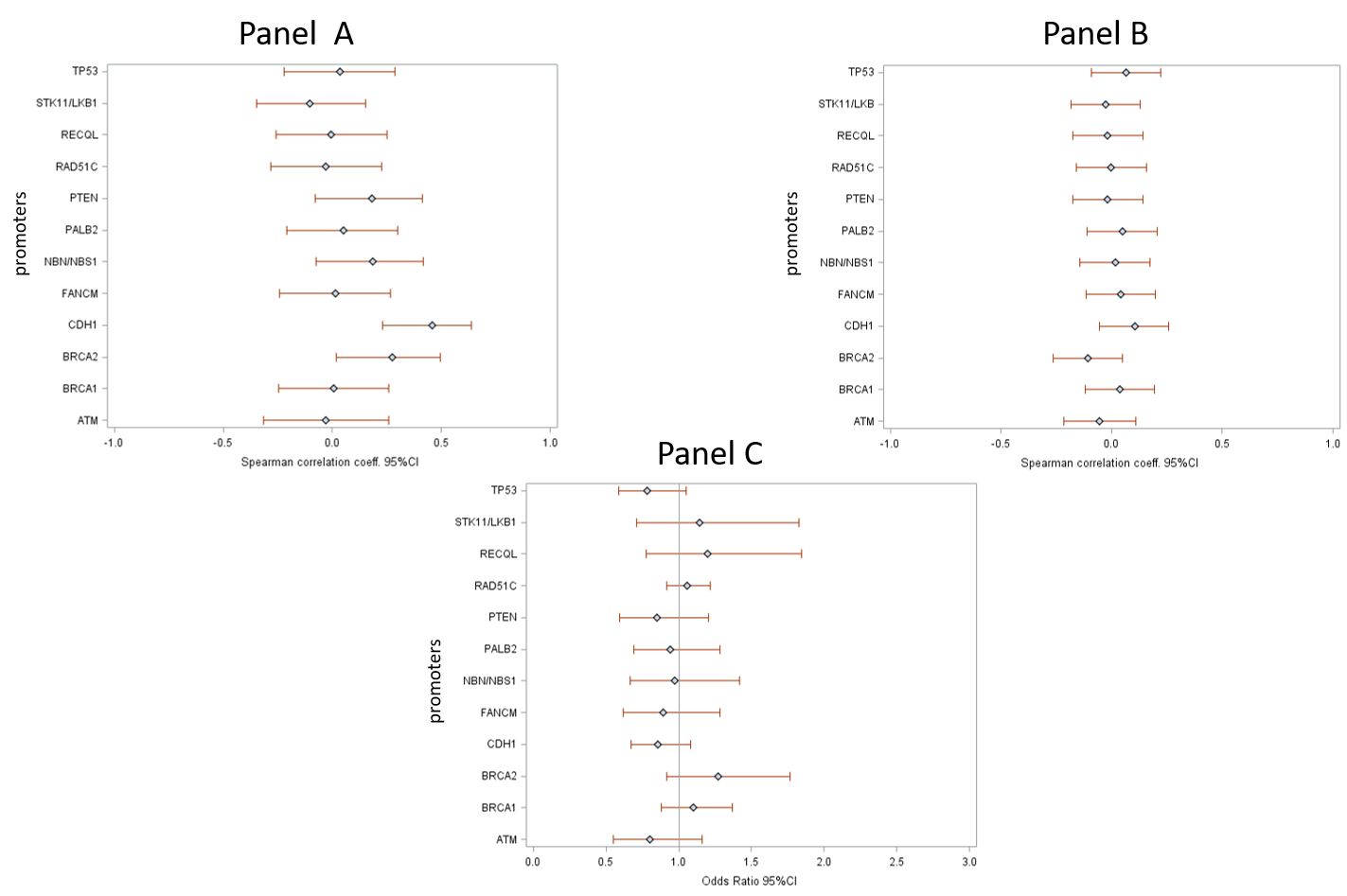


**Figure S1.** Evaluation of the relationship between methylation and age and between methylation and systemic treatments. Panel A and B report the Spearman correlation coefficient estimate (diamond) and its 95% Confidence Interval (CI) computed between age at blood withdrawal and methylation level of each promoter in controls (A) and cases (B). Panel C reports the odds ratio estimate (diamond) and the 95% CI obtained for each promoter from the univariate logistic model by considering the chemotherapy status at the time of blood withdrawal as a dependent variable (blood withdrawal during or after chemotherapy vs. no chemotherapy before blood withdrawal).

© 2018 by the authors. Licensee MDPI, Basel, Switzerland. This article is an open access article distributed under the terms and conditions of the Creative Commons Attribution (CC BY) license (http://creativecommons.org/licenses/by/4.0/).
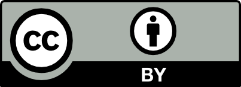

Supplement: Supplementary file 1 [file cancers-11-00058-s001.zip › cancers-417157-suppl-final/cancers-417157-suppl.-proof.docx]
